# A practical approach to programmatic assessment design

**DOI:** 10.1007/s10459-017-9756-3

**Published:** 2017-01-24

**Authors:** A. A. Timmerman, J. Dijkstra

**Affiliations:** 1 Department of Family Medicine, Maastricht University, P.O. Box 616, 6200 MD Maastricht, The Netherlands; 2 Academic Affairs, University Office, Maastricht University, P.O. Box 616, 6200 MD Maastricht, The Netherlands

**Keywords:** Assessment quality, Programmatic assessment, Instructional design, Quality assurance, Medical education

## Abstract

Assessment of complex tasks integrating several competencies calls for a programmatic design approach. As single instruments do not provide the information required to reach a robust judgment of integral performance, 73 guidelines for programmatic assessment design were developed. When simultaneously applying these interrelated guidelines, it is challenging to keep a clear overview of all assessment activities. The goal of this study was to provide practical support for applying a programmatic approach to assessment design, not bound to any specific educational paradigm. The guidelines were first applied in a postgraduate medical training setting, and a process analysis was conducted. This resulted in the identification of four steps for programmatic assessment design: evaluation, contextualisation, prioritisation and justification. Firstly, the (re)design process starts with sufficiently detailing the assessment environment and formulating the principal purpose. Key stakeholders with sufficient (assessment) expertise need to be involved in the analysis of strengths and weaknesses and identification of developmental needs. Central governance is essential to balance efforts and stakes with the principal purpose and decide on prioritisation of design decisions and selection of relevant guidelines. Finally, justification of assessment design decisions, quality assurance and external accountability close the loop, to ensure sound underpinning and continuous improvement of the assessment programme.

## Introduction

Recent discourse about what constitutes good assessment of performance has led experts to state, that assessment requires a programmatic approach consisting of a deliberate and arranged set of assessment activities (Knight 2000; Lew et al. 2002; Schuwirth et al. 2002; Schuwirth and van der Vleuten 2011a; van der Vleuten et al. 2012). These activities are not just a set of separate tests, but include also peripheral activities, such as item analysis for coherence of content of tests. The whole of assessment is being regarded as more than the sum of its parts. Hence, a single instrument approach will not be able to provide all the information needed for a comprehensive evaluation of competence (Schuwirth and van der Vleuten 2004; Dijkstra et al. 2010). Research on assessment of (medical) competence focuses increasingly on the top (‘does’) level of ‘Millers pyramid’, mainly in authentic tasks (Miller 1990; Vleuten et al. 2010). This has also contributed to a shift in current thinking from assessment methods to assessment programmes, as these tasks to be assessed have become increasingly complex integrating several competencies (van der Vleuten and Schuwirth 2005; Schuwirth and van der Vleuten, 2011b). A programme of assessment combines several assessment activities to reach an accurate judgment of competence that is robust and defensible (Dijkstra et al. 2012; Dijkstra 2014). A programmatic design approach is a comprehensive process that supports the overview of competencies being measured, compensates for deficiencies through combining several instruments, and frees time and space by combining information from different sources. The promises and resulting advantages of a programmatic approach are summarised in Table 1 (Dijkstra et al. 2010; van der Vleuten et al. 2012).

**Table 1:** Promises and advantages of a programmatic approach to assessment. Adapted from: Dijkstra et al. 2010; Van der Vleuten et al. 2012

Promises/purposes	Advantages
Overview of what is and what is not being measured	Promote the validity of content and prevent emphasis on easy-to –measure elements (over- and underrepresentation)
Compensation for deficiencies of instruments by strengths of other instruments	Diverse spectrum of complementary measurement instruments capturing competence as a whole
Matching instruments to free space and time for the assessment of other subjects	Increase efficiency by reducing redundancy in information gathering
Combine information from different sources (tests/instruments) in high-stakes assessment	Reach better-informed and highly defensible high-stakes decisions
Multiple individual assessment points that are maximally informative to the learning process	Optimise the learning function of assessment (assessment *for* learning)
Aggregated data used for high-stakes pass/fail and remediation decisions	Optimise the certification function (assessment *of* learning)
Reducing bias in assessment of complex tasks through smart sampling strategies and procedural measures	Expert judgment of competence in performing daily tasks becomes valid and reliable

A programmatic approach to assessment is holistic in nature and is not just about delivery of a test. In a programme of assessment, the implications need to be considered regarding faculty development, financial and organisational resources, and alignment with a specific educational context and curriculum. Limited sources are available that address assessment from a more comprehensive perspective. The Standards for Educational and Psychological Testing [(AERA), 2014] that cover a wide range of assessment activities, still have a focus on the development of single tests. Other approaches take a more quality perspective to describe programmes of assessment, e.g. Baartman et al. (2006). These quality frameworks do not provide guidance on how to design a programme of assessment and therefore Dijkstra et al. (2010, 2012) developed a framework for programmes of assessment and subsequently formulated guidelines for a programmatic design approach. The framework was derived from focus group discussions with international assessment experts in order to gain a comprehensive picture of the dimensions that needed to be covered (Dijkstra et al. 2010). The guidelines based on the framework were subsequently developed and validated through a structured interview approach with assessment experts (Dijkstra et al. 2012). The concomitant purpose was developing a common language in programmatic assessment. These studies resulted in a framework that contains 73 guidelines that cover six assessment programme dimensions, always related to the purpose of assessment, or the function it should fulfil: (1) The programme in action, which refers to the assessment activities as executed; (2) Supporting the programme, facilitating activities to achieve the purpose as good as possible with the current assessment practices; (3) Documenting the programme, which refers to describing the learning environment, content domain mapping and rules and regulations; (4) Improving the programme, by evaluation and development for future assessments, including change management; (5) Justification of the programme, mainly to external parties for acceptability and scientific underpinning; (6) The overarching dimensions Stakeholders and Infrastructure were added, since no programme functions in a contextual vacuum.

The guidelines are comprehensive and not bound to a specific educational context or approach. In comparison to studies describing quality criteria (Baartman et al. 2007) or tips for programmes of assessment (van der Vleuten et al. 2014), these guidelines are also not bound to a specific educational philosophy. There are no clear-cut criteria, or principles, that can be used as a recipe for a sound programme of assessment. The downside is that these 73 guidelines are less concrete and require a translation to educational practice, necessitating expert judgment to use these guidelines appropriately. As it is complex and demanding to keep an overview when applying these interrelated guidelines simultaneously, the goal of this study was, to develop a practical support for the application of a programmatic design approach to assessment. A postgraduate medical training programme was used as an educational context. This case is analysed in a qualitative manner by answering the following research question: What concrete steps need to be taken in the (re)design of an assessment programme using a programmatic approach?

## Methods

In previous research, a case study was performed to analyse the process of applying the 73 guidelines for programmatic assessment design to the running assessment programme of the Dutch Residency Training Programme in General Practice (RTPGP) (Dijkstra 2014). In the current study, the evaluation process of the RTPGP case was analysed and deconstructed. From this process analysis we derived a stepwise approach for (re-) designing a programme of assessment applicable to diverse educational settings.

### Case study

**Fig. 1:**
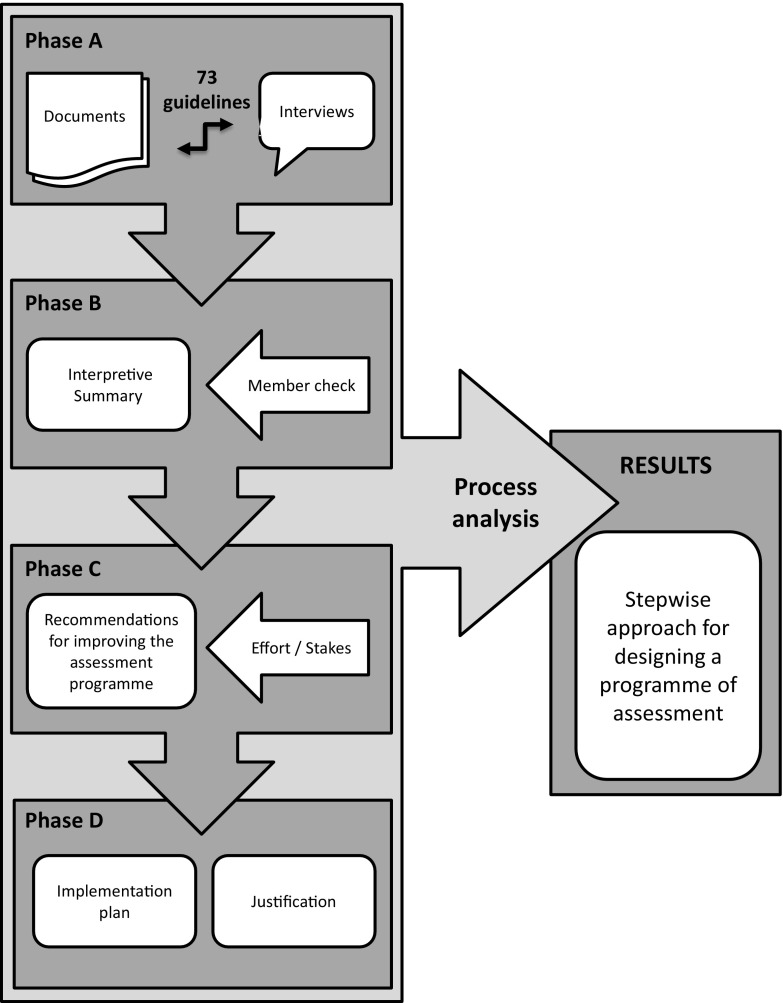
Methodological approach to case study, process analysis and deconstruction

The Dutch Residency Training Programme in General Practice (RTPGP), was purposefully selected, because of a well-described and documented competency based educational setting and assessment programme. The 3-year residency training consists of workplace-based learning in the authentic GP setting, as well as internships at a hospital, nursing home and psychiatric clinic. In addition, the residents attend to formal education 1 day per week. The assessment programme consists of assessments at all levels of Miller’s pyramid with a traditionally strong focus on observation in GP practice (Miller 1990). Assessment data are aggregated on the ComBel instrument by both GP supervisors and GP (or psychologist) trainers, which combines the outcomes of various test results and observations, to gain in‐depth information about the trainees’ achievement in each of the 7 CanMeds competencies every 3 months (Tromp et al. 2012). The programme director makes a summative decision about promotion to the next training year or graduation. A national assessment working group, representing the 8 Dutch RTPGP training programmes, governs assessment practices, through describing assessment principles, regulations, and instruments to be used. The aim of the evaluation of the RTPGP assessment programme was to identify areas for improvement and provide recommendations for redesign, including an implementation plan. As shown in Fig. 1 this process was divided in 4 phases, which will be further outlined here.

#### Phase A

The framework for programmes of assessment and associated 73 guidelines (Dijkstra et al. 2012) were used to evaluate the assessment of the RTPGP at Maastricht University. The evaluation was done based on two formal documents, that describe the vision statement and procedures of this assessment programme: ‘The national assessment plan’ (Dutch Institutes for General Practice 2011a) and ‘The national assessment protocol’ (Dutch Institutes for General Practice 2011b). Next to this document analysis, relevant stakeholders (e.g. assessment coordinator, teachers, and management) were interviewed to outline running assessment practices and gain input for further interpretation of the evaluation.

#### Phase B

A written interpretative summary of the evaluation (strengths and weaknesses) of the RTPGP assessment programme was drawn up by the authors. This summary was checked with the national assessment coordinator and the national assessment working group of the umbrella organisation of the Dutch RTPGP institutes for completeness and appropriateness.

#### Phase C

From the interpretive summary and input of the member check, a set of recommendations for the assessment redesign of RTPGP was formulated. These recommendations correspond to the sets of guidelines that need to be addressed in a coherent fashion when designing an assessment programme. The feasibility of these recommendations was determined based on the required effort for implementation as well as the associated stakes (or risks) and needed resources.

#### Phase D

Both from the priorities derived from the recommendations and the feasibility check an implementation plan was drawn up. This included a justification of needed design decisions based on best-practice and scientific evidence and concrete tips for change management, corresponding with the improving the programme dimension of the framework for programmatic assessment.

### Process analysis and deconstruction

As authors, we reflected on the phases and actions performed in the case study: from the evaluation of the RTPGP assessment programme to the formulation of the implementation plan. The questions and worries raised by the stakeholders during the interviews were taken as a starting point for deconstructing this process. During this analysis, our focus was on identifying the core elements that have characterised the evaluation of the RTPGP assessment programme. These core elements were translated in concrete actions, with the aim of achieving an approach to programmatic assessment design, a practical support which will be applicable across diverse educational contexts. In the remainder of this section the identified core elements will be outlined. The process of analysis and deconstruction is described, using the RTPGP case as a reference point.

#### Phase A

Characteristic for this evaluation phase was the focus on data collection. An iterative exploratory approach was used to navigate between the analysis of the formal documents, stakeholder interviews and guidelines. The biggest challenge was to keep overview of all assessment practices. Therefore interviewing the assessment coordinator, who was a linking pin between national assessment policy and local practices, was crucial to collect and interpret data on the running assessment programme. A key in this process was the confirmation of the *principle purpose* of the assessment programme, by explicitly addressing this in the interviews. How obvious this may seem, beliefs about the purposes of the assessment programme may not always be on the surface. In the case of RTPGP, the formative function is highly valued in running assessment practices; however, the summative ‘go/no go decision’ is the main purpose in the national assessment protocol.

#### Phase B

To write a summary report which is suitable for stakeholders involved with assessment, education, and management it was important to apply the framework and guidelines to the specific educational context (including jargon) of the RTPGP programme. The process of application consisted of acknowledging the context of the assessment programme: in the RTPGP case this was the specific relation between the national and local setting and workplace based learning with specific stakeholders—i.e. GP clinical supervisors, and identifying needs for development. In the RTPGP case it was prioritised to achieve balanced assessment contents to assure appropriate coverage of all competencies—i.e. the guidelines about domain mapping. In this phase, our goal was to reach a better fit of the applied (73) guidelines. Therefore, the guidelines needed to be re-grouped in order to clarify the relevance for this specific educational context. This re-grouping resulted in thematically ordered suggestions for improvement to (re-)design the assessment programme. For the RTPGP assessment programme, 12 themes of recommendations were formulated that need to be addressed in a programmatic (re-)design approach: 1. Purpose of the programme, 2. Resources/infrastructure, 3. Stakeholders, 4. Content and components, 5. Coherence and effects, 6. Decisions, standards and actions, 7. Robustness, 8. Context and implementation, 9. Procedures; rules and regulations, 10. Quality assurance, 11. Change management, and 12. Justification.

#### Phase C

The focus of this next phase was to determine the starting point for the improvement of the assessment programme. Simultaneously implementing 12 recommendations appeared to be not feasible and therefore some priorities needed to be set. Essential to prioritizing the recommendations was central governance of the programme, to keep overview and avoid counterproductive measures implemented at the same time. In the case of RTPGP the national assessment working group was fit for this job, as it consists of the 8 local assessment coordinators. This body is responsible for setting out an assessment scheme that includes several assessment instruments, and its transposition into the local setting, including the alignment of decisions. (Dutch Institutes for General Practice 2011a) Balancing effort and stakes was only possible when including the perspectives of all stakeholders. Therefore the priority of improvements was determined in a team effort of the eight RTPGP institutes. This was secured during a meeting with the national assessment working group, in which the strengths and weaknesses in the interpretive summary and needed changes in assessment practices were discussed. Mutual recognition of the perceptions of different assessment coordinators was created, and as such enriched the *national* design decisions with a support base for *local* acceptance of the implementation plan.

#### Phase D

In this phase, the justification and underpinning of the proposed improvements of the assessment programme were essential, to gain acceptance of the implementation plan. The national assessment coordinator and national assessment working group are responsible for identifying and documenting evaluation criteria and develop an implementation plan in advance. The eight RTPGP training institutes independently implement the needed changes in their own context (Dutch Institutes for General Practice 2011b). A local assessment coordinator serves as ‘linking pin’, being indispensable for the identification of relevant contextual issues in change management and coaching the implementation process of assessment design decisions. At a later stage, these stakeholders have an important role in the evaluation whether the assessment design decisions have had the intended effect on the quality of the assessment programme. A pitfall for RTPGP is not to close the feedback loop for quality assurance when the re-designed assessment programme is running. The national assessment working group seems to be the most suitable platform for this evaluation, both from the perspective of connecting diverse assessment contexts and respective responsibilities of participating stakeholders in the development and application of assessment activities.

## Results

From applying the 73 guidelines to the case of RTPGP, and the subsequent process analysis, four guiding steps in a programmatic approach were identified. These steps need to be covered in designing a programme of assessment:

A. Evaluate the current assessment practice and identify the needs for improvement.
B. Contextualise the framework and guidelines to fit the assessment environment.
C. Prioritise the needed changes in assessment by selecting relevant guidelines depending on effort and stakes.
D. Justify design decisions and develop an implementation plan.

Within each of these steps we describe actions to be taken, that support (re)designing an assessment programme in any given educational context. In total 12 actions are described in Table 2, which will be further outlined under the four respective steps in this results section.

**Table 2:** Stepwise approach for designing an assessment programme

*Step A. Evaluate the current assessment practice and identify needs for improvement*
1. Collect evaluation data in an iterative way: Combine formal assessment documents and interview stakeholders about current assessment practices
2. Keep an overview of the big picture by using sufficiently broad information including the assessment infrastructure: stakeholders’ expertise and roles, educational curriculum, stakes, resources, and legislation
3. Confirm the principal purpose of the assessment programme and ensure it is clearly formulated. Evaluate whether this is being adequately shown in actual assessment practices
*Step B. Contextualise the framework and guidelines to fit the assessment environment*
4. Use data collected on guidelines in the programme in action and documenting the programme dimensions as a contextual frame of reference for outlining the assessment environment
5. Organise several meetings and involve key stakeholders with assessment expertise to prepare an analysis of strengths and weaknesses of current assessment practices for identification of developmental needs
6. Re-order the guidelines to suit the context which should be guiding (instead of the framework) in a programmatic design approach
*Step C. Prioritise the needed changes in assessment depending on effort and stakes*
7. Translate identified needs into related investments (i.e. infrastructure, expertise, finances) and balance these with the stakes and principal purpose of the assessment programme
8. Data collected on guidelines in the dimensions supporting and improving the programme need to be used to reach consensus about the prioritisation of needed changes
9. Take an iterative approach when applying the selected guidelines, develop a working strategy involving key stakeholders to support central governance and avoid inefficient or counterproductive changes
*Step D. Justify design decisions and develop an implementation plan*
10. Document design decisions in a consistent way, taking into account legal regulations preferably based on scientific and/or at least practice based evidence
11. Develop a clear and concise implementation plan, including faculty development, to foster acceptability in the assessment context and serve external accountability
12. Take care that the feedback loop is being closed and schedule regular evaluation meetings, based on evaluation criteria in the implementation plan

### A. Evaluate the current assessment practice and identify needs for improvement

The overview of the current assessment practice needs to be clear, before being able to identify needs for improvement. There are two possible sources of information that can be used for clarification. Action 1 consists of firstly analysing formal assessment documents, describing e.g. principles of assessment and criteria, methods and tools used, and an educational blueprint. Secondly, interviewing key stakeholders with expertise and knowledge of the local assessment environment, to get inside knowledge and understand how the assessment is being carried out. This should lead to an iterative approach in which the formal documents provide structure and boundaries, while the interviews clarify the daily assessment practices in a specific educational setting. All decisions related to the design should be guided by the principal purpose of the assessment programme. As action 2 this requires that assessment information is sufficiently broad and provides a clear overview of available resources: stakeholders, stakes and finances, besides legislation and oversight of the educational curriculum. Current assessment activities are an important source of information on stakeholder roles, responsibilities, and tasks that need to be carried out. Action 3 is then to get clarity on the principal purpose at an early stage with broad stakeholder support, as it sets the roadmap for the programmatic approach. To decide on the principal purpose of an assessment programme the main question to ask is: *‘What is the essential activity (e.g. taking a summative decision) in the assessment programme?’* This principal purpose is often directly related to the *programme in action* dimension of the overarching framework. A clear goal facilitates achieving the optimal compromises in the assessment programme, balancing the requirements of assessment against possibilities of the environment in the most effective way. Without a clearly formulated principal purpose, design decisions can become ambiguous and may result in inconsistencies when designing the programme.

### B. Contextualise the framework and guidelines to fit the assessment environment

Knowledge of the (educational) context is required for developing an acceptable and feasible assessment programme, which does not function in a vacuum. Specific (local) educational issues, contemporary trends in education or the political environment may lead to differences in appreciated importance of certain guidelines in design decisions. In action 4 guidelines referring to the *programme in action* and *documenting the programme* dimensions serve as a frame of reference, combined with the information collected during the evaluation of the current assessment practice. A group of stakeholders knowledgeable of the educational context and assessment practices needs to be identified and interviewed to get a clear overview of contextual issues that influence assessment design, which is action 5. The added value of organising several team meetings with these stakeholders is that they may deliver valuable input for an analysis of strengths and weaknesses of the current assessment practice and serve as a member check for the identification of developmental needs. More specifically, these issues often arise in management board meetings or the media, and should be addressed appropriately in a programmatic design approach. Early identification of key stakeholder groups (e.g. teachers, students, programme directors) allows designers to (1) take multiple interests into account, (2) define respective roles and responsibilities in the design process and (3) support the (future) role of stakeholders in accepting the development and implementation of a programmatic approach. Assessment design is not only a psychometric measurement problem, but also an instructional design problem and even an organisational problem. Hence, it appeals to a broad spectrum of expertise that is often not present in one stakeholder alone, which makes the assessment design necessarily a team effort. In a programmatic design approach, diverse stakeholders bring in knowledge from their own fields of expertise to develop a defensible assessment programme that aligns with educational practice. The guidelines for programmatic assessment are no recipe for developing the single best assessment programme. Their relevance and applicability are contingent upon many contextual factors (e.g. resources, politics, stakeholders, educational goals) of the assessment environment. The framework defined by Dijkstra et al. (2010) showcases just one way of organizing the guidelines (Dijkstra et al. 2010). Action 6 is to re-interpret this theoretical exercise of ordering the guidelines for each specific context. Although in the RTPGP case study a starting point and way of applying the guidelines is suggested, there is no fixed order in which to apply them. Extensive knowledge about the local context is not only required to be able to decide on the relevance of guidelines, but also to determine which assessment activities are interrelated in assessment design. A team discussion aimed at achieving consensus with relevant stakeholders may support this process by developing a shared model of re-ordered guidelines for the specific educational context, as the basis for identifying developmental needs.

### C. Prioritise the needed changes in assessment depending on efforts and stakes

A potential pitfall is that most resources and efforts are spent on assessment activities that are easy to implement as they seem more appealing, at the expense of difficult assessment activities which often involve a higher stake. Action 7 is twofold: Firstly, checking available resources beforehand, which will allow compromises with regard to the scope of the assessment and/or decisions on extra investment. A clear overview of available resources before starting the design will serve the quality of the assessment programme: it sets the boundaries in advance and avoids disappointment about the feasibility of design decisions. Moreover, it allows for (more) efficient alternative assessment activities to be sought at an early stage. Resources refer not only to financial means, but also to expertise, time and infrastructure. Secondly, the identified developmental needs should to be translated into design decisions and related investments. Balancing efforts against stakes should be taken as a rule of thumb, which permeates all levels of assessment, ranging from the design of a programme or single assessment instrument to the act of writing items. Balancing the needed investment of resources with the gained output in achieving the purpose, echoes the framework’s first general guideline (Dijkstra et al. 2012). Action 8 is to prioritise in the initial design approach and decide what the premises are for selecting those guidelines relevant for programmatic assessment within this specific educational context. The evaluation of the current assessment programme on guidelines related to the dimensions *supporting* and *improving the programme*, probably contain valuable information to determine on priorities (e.g. taking robust summative decisions). These guidelines may be used to reach consensus on the needed changes and as a cross-check when developmental needs have been identified accurately. Action 9 requests an iterative approach commuting between guidelines, principal purpose and balancing of efforts and stakes, that aids in developing a working strategy for assessment design planning. In this process, central governance of assessment (design) is required to prevent contradictory decisions over time and between different activities. In the course of the development process, multiple experts (stakeholders) leave their imprints on assessment design. An oversight of the assessment programme serves a balanced use of available resources, careful planning of assessment activities and appropriate coverage of the educational and assessment purposes. This aids in preventing over- and underrepresentation of assessment activities.

### D. Justify design decisions and develop an implementation plan

To be able to evaluate the robustness of design decisions, the reasons for these choices should be clarified and documented, which is action 10. Decisions should preferably be based on scientific evidence, however, since not all assessment activities are sufficiently researched, best practices are a viable alternative. Sound underpinning of design decisions and explicit enunciation of their underlying rationale, can support acceptance by stakeholders. In the design process this should be actively fostered and therefore relations with the future work domain are of paramount importance. This action refers to dimensions of *justifying and improving the programme* from the overarching framework. The need for change (i.e. a design decision) has to be sufficiently clear for all stakeholders. A common pitfall is that alignment of decisions is only considered in the initial stages of assessment development. Afterwards, when the assessment programme is running, alignment of assessment activities remains an essential quality assurance procedure. Therefore it is crucial that the expertise of faculty (to be) involved in the running assessment programme is assured. By making purposeful and balanced decisions a team effort, as stated under actions 5 and 9, it becomes easier to support external accountability for the programme. Action 11 is developing an implementation plan with unambiguous and agreed evaluation criteria, crucial for a successful implementation of an assessment programme, in which principles of change management play a vital part. Therefore, finding the person who is fit and responsible for this job in each particular assessment environment is necessary. In small organisations appointment of just one person may suffice, in larger ones a team may be more appropriate. In action 12 assessment decisions and their underpinnings should be revisited at a later stage, to stimulate continuous learning and improvement—i.e. closing the loop. It is recommended that an organisation keeps records of past trajectories, to avoid counteractive decisions and re-invention of ‘the wheel’ in the future and schedule regular evaluation meetings when the (re-) designed assessment programme is up and running. Evidence for quality assurance of the assessment programme can be gathered from different sources (e.g. expert panels, work field). External parties (e.g. the public, government) are increasingly requesting accountability for running assessment practices, which can be raised by defining external stakeholders and inviting external review panels (e.g. accrediting organisations) dedicated to evaluating the programme.

## Discussion

The goal of this study was to develop a practical support for a programmatic design approach to assessment and to determine the steps that need to be taken in the (re)design of an assessment programme. The process analysis of the RTPGP case, evaluated by applying 73 guidelines for programmatic assessment design (Dijkstra 2014), has led to the identification of four steps that need to be taken in the (re)design of an assessment programme: evaluation, contextualisation, prioritisation, and justification (see Table 2). Through identifying these steps, substantiated with 12 actions, the application of the framework for designing programmes of assessment and related 73 guidelines, is supported and made more concrete and feasible. The 12 actions outlined serve as a road map to describe, evaluate, justify and improve the quality of an assessment programme, where the framework and guidelines only provide a vocabulary (Dijkstra 2014).

In formulating a stepwise approach to programmatic assessment design, we aim to contribute to fulfil the stated promises of a programmatic approach to assessment (see Table 1). These promises do align with the functions that need to be united within an assessment programme: facilitating learning processes (assessment *for* learning), maximising the robustness of high stakes decisions (assessment *of* learning on promotion/selection of learners) and providing information for improving instruction and the curriculum (van der Vleuten et al. 2012). Preconditions for fulfilling these functions are creating an overview of all assessment activities, alignment of complementary instruments capturing competence as a whole, minimising redundancy of assessment components in the programme and optimising the accountability of running assessment activities (Dijkstra et al. 2010; van der Vleuten et al. 2012). These preconditions are also reflected in the identified steps and actions in our study. The overview of all assessment activities is encouraged through *contextualisation* by using sufficiently broad information of the current assessment programme (action 2), organising several meetings with key stakeholders to analyse its’ strengths and weaknesses (action 5) and develop a working strategy to support central governance (action 9). The alignment of complementary instruments and minimising redundancy of assessment components is addressed in the step *prioritisation* of the needed changes depending on effort and stakes (action 7 and 8), and development of a working strategy that avoids inefficient and counterproductive changes (action 9). In the *justification* of design decisions accountability of running assessment practices is promoted by concise and persistent documentation while taking into account legal regulations (action 10). Clear and acceptable procedural measures in the implementation plan may support this process in an iterative manner to reduce bias in assessment of complex tasks (action 11). From a utilitarian perspective the quality of assessment is always being defined in terms of fitness-for-purpose (action 3) in the specific educational context (Dijkstra 2014).

### Strengths and limitations

One strength of the four steps is that these are not limited to a specific (educational) paradigm. Therefore they are applicable in a wide range of educational contexts. The case study provides examples of the RTPGP case that illustrate the abstract stepwise approach for the diverse stakeholders involved in assessment practices. A potential source of bias is that we as researchers conducted both the case study and process analysis. This may have impacted on internal validity of the study results. However, our reflection in terms of an in depth analysis was needed to identify the core elements of the conducted evaluation approach. We do realise that the developed stepwise approach was based on one case study in a postgraduate medical context. We believe in the applicability to other educational settings, but we do encourage others to prove this in future studies and practice.

### Implications for future research

Although few examples of assessment programmes (Bok et al. 2013; Dannefer and Henson 2007) for a specific educational context have been identified, there is no concrete evidence supporting the practical value of a programmatic design approach available yet. Future research can address the issue of transferability by applying the four steps in diverse educational contexts. Evaluating the impact of assessment programmes in terms of achieving their main purpose will be an important step in validating the guidelines for programmatic assessment and related design approach. Besides studying the transferability of the stepwise approach itself, application in diverse educational settings will also provide us with examples of potential compromises on quality characteristics for any assessment method: reliability, validity, educational impact, acceptability and costs depending on purpose and specific assessment context (van der Vleuten 2016). We trust that future research will substantiate and replace the promises of a programmatic approach to assessment with evidence for the stated advantages of programmatic assessment.

## Conclusion

The outline of a stepwise approach to programmatic assessment design is intended to help educators make appropriate design decisions for their specific educational context. The involvement of context expertise enables to broaden the scope ‘from assessment methods to assessment programmes’ and to consider assessment not only as a psychometric problem, but rather as both an instructional and organisational design problem: through first asking what the essential activity is in the assessment programme, the balancing of the efforts (i.e. resources) with the stakes is supported, while fostering a constructive alignment with the educational curriculum. This study provides a stepwise approach to apply a theory based model for programmatic assessment design. This approach and the model need implementation in other educational settings as a route to scientific validation and further development of a practice based design approach, applicable in diverse educational contexts in a feasible and pragmatic manner.
